# Fungal gene expression levels do not display a common mode of distribution

**DOI:** 10.1186/1756-0500-6-559

**Published:** 2013-12-28

**Authors:** Minou Nowrousian

**Affiliations:** 1 Lehrstuhl für Allgemeine und Molekulare Botanik, Ruhr-Universität Bochum, 44780 Bochum, Germany

**Keywords:** RNA-seq, Fungi, Gene expression distribution, Bimodal distribution, Zipf’s law

## Abstract

**Background:**

RNA-seq studies in metazoa have revealed a distinct, double-peaked (bimodal) distribution of gene expression independent of species and cell type. However, two studies in filamentous fungi yielded conflicting results, with a bimodal distribution in *Pyronema confluens* and varying distributions in *Sordaria macrospora*. To obtain a broader overview of global gene expression distributions in fungi, an additional 60 publicly available RNA-seq data sets from six ascomycetes and one basidiomycete were analyzed with respect to gene expression distributions.

**Results:**

Clustering of normalized, log_2_-transformed gene expression levels for each RNA-seq data set yielded distributions with one to five peaks. When only major peaks comprising at least 15% of all analyzed genes were considered, distributions ranged from one to three major peaks, suggesting that fungal gene expression is not generally bimodal. The number of peaks was not correlated with the phylogenetic position of a species; however, higher filamentous asco- and basidiomycetes showed up to three major peaks, whereas gene expression levels in the yeasts *Saccharomyces cerevisiae* and *Schizosaccharomyces pombe* had only one to two major peaks, with one predominant peak containing at least 70% of all expressed genes. In several species, the number of peaks varied even within a single species, e.g. depending on the growth conditions as evidenced in the one to three major peaks in different samples from *Neurospora crassa*. Earlier studies based on microarray and SAGE data revealed distributions of gene expression level that followed Zipf’s law, i.e. log-transformed gene expression levels were inversely proportional to the log-transformed expression rank of a gene. However, analyses of the fungal RNA-seq data sets could not identify any that confirmed to Zipf’s law.

**Conclusions:**

Fungal gene expression patterns cannot generally be described by a single type of distribution (bimodal or Zipf’s law). One hypothesis to explain this finding might be that gene expression in fungi is highly dynamic, and fine-tuned at the level of transcription not only for individual genes, but also at a global level.

## Background

The availability of transcriptomics techniques to determine gene expression not only allows the parallel analyis of expression of many individual genes, but also prompted the question whether genome-wide expression patterns follow specific distributions [1-3]. If there were such global distributions common to many biological systems, they could reveal higher-level mechanisms underlying gene expression, and could potentially be used, for example, to predict cellular reactions at systems level, or for quality control and normalization of transcriptomics data sets [2,4,5]. Early analyses were based on SAGE (serial analysis of gene expression) or microarray data, mostly of *Escherichia coli*, the yeast *Saccharomyces cerevisiae*, and metazoa including humans. These studies mostly found power law distributions or combinations of log-normal and power law distributions [1-6]. Power law distributions tended to follow Zipf’s law, i.e. the log-transformed expressions of the investigated genes were inversely proportional to the log-transformed ranks of expression.

In recent years, RNA-seq analysis has replaced microarrays for most transcriptomics analyses [7,8]. A study of gene expression distributions of log-transformed RPKM (reads per kilobase per million mapped reads) values from several metazoa revealed a bimodal distribution that could be described as the sum of two normal distributions (a high-expression and a low-expression peak) [9]. The authors suggest that mRNAs falling within the high-expression peak constitute the active part of the transcriptome, whereas the mRNAs of the low-expression peak might be the result of “leaky” transcription not leading to functional mRNAs. A similar observation was also made in a study of chimeric transcripts in humans. These transcripts were predominantly found in the low-expression peak of a bimodal distribution [10]. Possible reasons why this bimodal distribution was not observed previously might be the higher sensitivity of RNA-seq which allows better resolution for weakly expressed genes, differences in data processing and plotting methods, and the type of cells and organisms that were analyzed [9].

However, as these RNA-seq-based studies were conducted with data sets from metazoa, it was not clear whether a bimodal distribution was restricted to this group or a general feature of gene expression in a wider range of organisms. Two analyses of gene expression distribution in filamentous fungi based on RNA-seq data did not yield conclusive results with regard to this question. In a study with *Sordaria macrospora*, distributions from four different conditions resulted in distributions with two or three peaks [11], whereas the analysis of three different conditions in *Pyronema confluens* gave distributions with three peaks, two of which contained the majority of all genes, thereby resembling a bimodal distribution [12]. To more comprehensively address the question whether fungal gene expression can be described by a bimodal distribution, in this study an additional 60 publicly available RNA-seq data sets from six ascomycetes and one basidiomycete were analyzed.

## Results and discussion

### Fungal gene expression distributions are not generally bimodal

**Figure 1:**
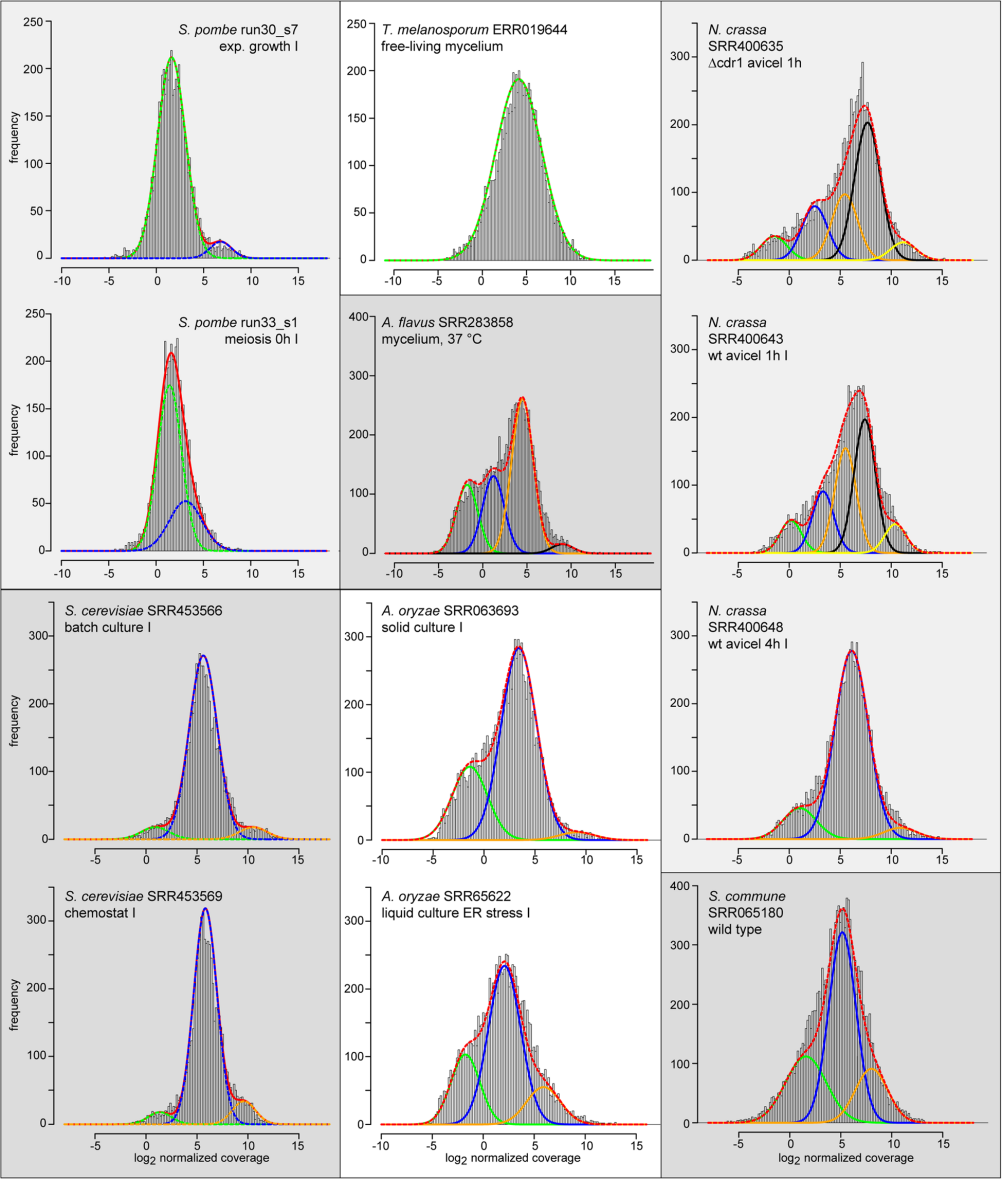
**Distribution of gene expression levels for 12 RNA-seq data sets from seven fungal species.** Histograms of normalized, log_2_-transformed coverage for each locus tag (grey bars), and estimated frequency distributions. Locus tags without coverage were not included in this analysis. The distribution function (red line) for each data set was dissected into components (blue, green, yellow, orange, and black lines) that are normal distributions with varying means and variances that make up different proportions of the observed distribution (Additional file 3: Table S1). Histograms and frequency distributions for the other data sets that were analyzed in this study can be found in Figures S1-S11 in Additional file 3.

RNA-seq data sets from 60 individual experiments were downloaded from public databases (Table 1, Additional file 1). These included data sets from the ascomycetes *Schizosaccharomyces pombe*, *S. cerevisiae*, *Tuber melanosporum*, *Neurospora crassa*, *Aspergillus flavus*, and *Aspergillus oryzae*, and the basidiomycete *Schizophyllum commune*[13-19]. Sequence reads were quality-trimmed, and mapped to the annotated mRNAs or coding sequences from the corresponding species. Only reads that mapped in their entire lengths to a single locus tag were used for downstream analysis. Mapped bases per locus tag were counted (coverage), normalized within the data set for each species, and log_2_-transformed coverage values were used for downstream analysis (Additional file 2). Figure 1 shows histograms of coverage values for 12 selected RNA-seq data sets, histograms for all data sets are given in Additional file 3: Figures S1-S11. This visualization already revealed differences between species, and also between different data sets from one species, e.g. in the case of *N. crassa*, where growth on cellulose (avicel) for 1 h resulted in a distinctly left-skewed distribution, whereas the left tail was much less pronounced after 4 h on avicel (Figure 1).

**Table 1:** Summary of datasets that were analyzed for overall transcriptome patterns

**Sample**	**Condition**	**No. of peaks**	**Main peaks**^**1**^
**Taphrinomycotina**			
*S. pombe* run30_s7	YE medium, exponential growth	2	1
*S. pombe* run30_s8	YE medium, exponential growth	2	1
*S. pombe* run33_s1	meiosis, 0 h	2	2
*S. pombe* run33_s2	meiosis, 0 h	2	2
*S. pombe* run33_s3	meiosis, 1 + 2 h	2	2
*S. pombe* run33_s4	meiosis, 1 + 2 h	2	2
*S. pombe* run33_s5	meiosis, 3 + 4 h	2	2
*S. pombe* run33_s6	meiosis, 3 + 4 h	2	2
*S. pombe* run33_s7	meiosis, 5 + 6 h	2	2
*S. pombe* run34_s1	meiosis, 5 + 6 h	2	2
*S. pombe* run34_s2	meiosis, 7 + 8 h	2	2
*S. pombe* run34_s3	meiosis, 7 + 8 h	2	2
**Saccharomycotina**			
*S. cerevisiae* SRR453566	respiro-fermentative (batch)	3	1
*S. cerevisiae* SRR453567	respiro-fermentative (batch)	3	1
*S. cerevisiae* SRR453568	respiro-fermentative (batch)	3	1
*S. cerevisiae* SRR453569	fully respiratory (chemostat)	3	1
*S. cerevisiae* SRR453570	fully respiratory (chemostat)	3	2
*S. cerevisiae* SRR453571	fully respiratory (chemostat)	3	1
**Pezizomycetes**			
*P. confluens* GSM1020388/89	light grown, sexual mycelium	3	2
*P. confluens* GSM1020390/91	dark grown, vegetative mycelium	3	2
*P. confluens* GSM1020392/93	vegetative mycelium mix	3	2
*T. melanosporum* ERR019644	free-living mycelium	1	1
*T. melanosporum* ERR019645	fruiting bodies	3	1
*T. melanosporum* ERR019646	ectomycorrhizal root tips	3	1
**Sordariomycetes**			
*N. crassa* SRR400635	Δcdr1, avicel, 1 h	5	3
*N. crassa* SRR400636	∆cdr1, avicel, 4 h	4	2
*N. crassa* SRR400637	∆cdr1, sucrose, 1 h	5	3
*N. crassa* SRR400638	∆cdr1, sucrose, 4 h	5	3
*N. crassa* SRR400639	∆cdr2, avicel, 1 h	5	3
*N. crassa* SRR400640	∆cdr2, avicel, 4 h	3	3
*N. crassa* SRR400641	∆cdr2, sucrose, 1 h	5	3
*N. crassa* SRR400642	∆cdr2, sucrose, 4 h	5	3
*N. crassa* SRR400643	wild type, avicel, 1 h	5	3
*N. crassa* SRR400644	wild type, avicel, 1 h	5	3
*N. crassa* SRR400645	wild type, avicel, 1 h	5	3
*N. crassa* SRR400646	wild type, avicel, 2 h	5	3
*N. crassa* SRR400647	wild type, avicel, 0.5 h	5	3
*N. crassa* SRR400648	wild type, avicel, 4 h	3	1
*N. crassa* SRR400649	wild type, avicel, 4 h	3	2
*N. crassa* SRR400650	wild type, avicel, 4 h	3	2
*N. crassa* SRR400651	wild type, no carbon, 1 h	5	2
*N. crassa* SRR400652	wild type, no carbon, 4 h	3	3
*N. crassa* SRR400653	wild type, no carbon, 4 h	2	2
*N. crassa* SRR400654	wild type, no carbon, 4 h	3	3
*N. crassa* SRR400655	wild type, sucrose, 1 h	3	2
*N. crassa* SRR400656	wild type, sucrose, 1 h	5	3
*N. crassa* SRR400657	wild type, sucrose, 1 h	5	3
*N. crassa* SRR400658	wild type, sucrose, 4 h	5	3
*N. crassa* SRR400659	wild type, sucrose, 4 h	5	3
*N. crassa* SRR400660	wild type, sucrose, 4 h	5	3
*S. macrospora* GSM832529	wild type vegetative mycelium	2	2
*S. macrospora* GSM832533	wild type vegetative mycelium	2	2
*S. macrospora* GSM832530	wild type sexual mycelium	3	1
*S. macrospora* GSM832534	wild type sexual mycelium	2	2
*S. macrospora* GSM832531	wild type protoperithecia	3	3
*S. macrospora* GSM832532	wild type protoperithecia	3	3
*S. macrospora* GSM832535	pro1 protoperithecia	3	2
*S. macrospora* GSM832536	pro1 protoperithecia	3	2
**Eurotiomycetes**			
*A. flavus* SRR283857	30°C	3	2
*A. flavus* SRR283858	37°C	4	3
*A. oryzae* SRR043191	solid culture	3	2
*A. oryzae* SRR035603	solid culture	3	2
*A. oryzae* SRR043192	liquid culture	4	3
*A. oryzae* SRR063693	liquid culture	3	2
*A. oryzae* SRR043193	solid culture ER stress	4	3
*A. oryzae* SRR065622	solid culture ER stress	3	3
*A. oryzae* SRR043194	liquid culture ER stress	4	3
*A. oryzae* SRR065623	liquid culture ER stress	3	2
**Basidiomycota**			
*S. commune* SRR065180	wild type	3	3
*S. commune* SRR065181	∆hom2	3	3
*S. commune* SRR065182	∆fst4	3	3

∆, Delta symbol denotes deletion mutants. For each analyzed sample, species and accession number are given. Data for *P. confluens* and *S. macrospora* were analyzed for expression distributions in previous studies with GEO accession numbers GSE41631 and GSE33668, respectively [11,12]; the other datasets were analyzed in this study. For *P. confluens*, data from independent biological repetitions were subjected to a combined analysis, but results were similar to analyses of the individual datasets [12]. For all other analyses, data from individual RNA-seq experiments were used. RNA-seq data sets from the following studies were used for this analysis: *Schizosaccharomyces pombe*[18], *Saccharomyces cerevisiae*[14], *Pyronema confluens*[12], *Tuber melanosporum*[16], *Neurospora crassa*[13], *Sordaria macrospora*[11], *Aspergillus flavus*[19], *Aspergillus oryzae*[17], *Schizophyllum commune*[15]. More information on the studies and reference genomes used for mapping can be found in Additional file 1.

^1^proportion ≥ 0.15 (15%).

The gene expression distributions then were clustered to determine whether they could be dissected into distinct normal distributions (see Additional file 3: Method S1). The results confirmed the rather large variations between and within species, with one to five peaks (individual normal distributions) distinguishable. Even considering only peaks that contained a proportion of at least 15% of the clustered genes resulted in a range of one to three main peaks (Figure 1, Table 1, Additional file 3: Table S1 and Figures S1-S11), indicating that gene expression distributions are not generally bimodal in fungi.

**Figure 2:**
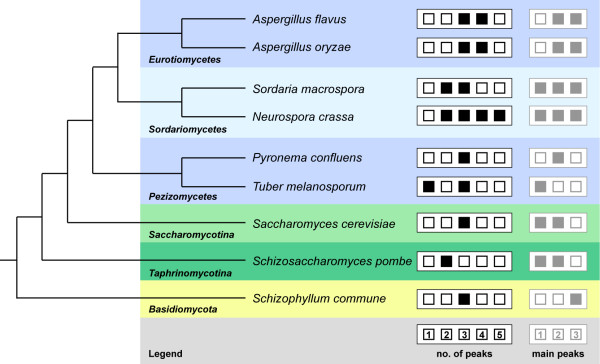
**Phylogenetic relationships of the species included in this analysis.** The species tree is derived from [12]. On the right, the number of peaks observed in the expression distributions for this species is indicated as filled black boxes, the number of major peaks (≥ 15% of genes in this peak) is indicated as filled gray boxes.

The fungal species analyzed in this study represent a wide phylogenetic range, and one might wonder whether the number of peaks in gene expression distributions is correlated with the phylogenetic position of the investigated species. However, there does not seem to be any obvious correlation, because specific numbers of peaks or main peaks were not consistently associated with certain phylogenetic groups (Figure 2). An exception might be the two unicellular yeasts *S. cerevisiae* and *S. pombe*, both of which have at most two main peaks, with one of the main peaks consistently containing at least 70% of all analyzed genes (Additional file 3: Table S1). A single dominant main peak is also observed in the early-diverging filamentous ascomycete *T. melanosporum* (Additional file 3: Figure S4); however, in this case it might be due to the fact that the analyzed samples contained cells from different tissue types with possibly different expression patterns for a number of genes [16]. The resulting mixing of gene expression distributions could lead to a unimodal distribution [9,20].

It was predicted from simulations of mixtures of different cell types as well as analysis of expression data from the fly *Drosophila melanogaster* that mixing can lead to deviations from the bimodal distribution not only towards unimodal, but depending on the conditions towards left-skewed, sometimes multimodal distributions [20]. Therefore, one might ask whether analyzing mycelia that comprise different cell types might also result in the multi-peaked distributions observed in several fungal data sets. However, while this explanation might be true for some of the datasets, it does not fit the case of *N. crassa*, where vegetative mycelia were grown for 16 h, and then subjected to different treatments for 0.5-4 h [13]. Under these conditions, only vegetative hyphae can develop that do not contain many different cell types, and any differences in distributions observed after the short treatments are unlikely to be due to the development of novel cell types. For example, growth on cellulose (avicel) resulted in a five-peaked distribution in early time points (0.5-2 h), but only three peaks were observed after 4 h. Five and three peaks were also found after 1 h and 4 h without carbon source, respectively, whereas growth in sucrose lead to overall similar distributions after 1 and 4 h (Table 1, Additional file 3: Figures S6, S7, and S8). Thus, the differences are most likely not due to developmental changes within the observed time frame; rather, *N. crassa* seems to be able to quickly shift its global gene expression as a reaction to changes in external conditions. This might also be the case in *A. flavus*, where cultures were grown for 24 h under identical conditions except for the growth temperature [19], leading to a more strongly left-skewed distribution with a higher number of peaks at 37°C compared to 30°C (Table 1, Additional file 3: Figure S9).

Another reason for failing to detect peaks might be a lack of resolution caused by insufficient coverage, especially for weakly expressed genes [9]. To determine whether the number of observed peaks was depending on the base coverage, the number of peaks and main peaks was plotted against the counted bases for each RNA-seq data set (Additional file 3: Figure S12). However, no clear correlation was found, indicating that coverage was not a critical factor in this analysis.

One might also ask whether the number of peaks that can be detected is correlated with the number of genes or the size of the genome of the organism under investigation. However, plotting of peak numbers versus the number of genes or genome size did not show any significant correlation (Additional file 3: Figure S13).

### Fungal gene expression distributions do not generally follow Zipf’s law

**Figure 3:**
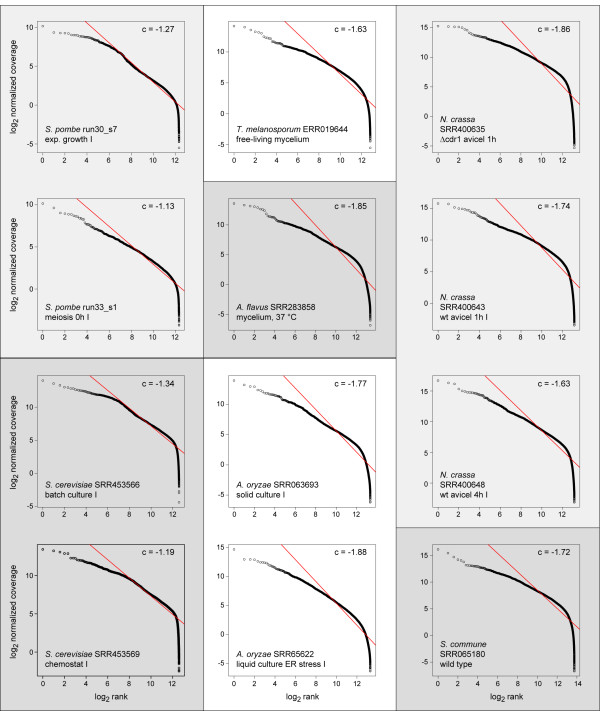
**Test for Zipf’s law in distributions of gene expression levels for 12 RNA-seq data sets from seven fungal species.** Each data set was sorted independently by normalized log_2_-transformed coverage, and log_2_-transformed coverage was plotted against the log_2_-transformed expression rank for each locus tag. Locus tags without coverage were not used in this analysis. A linear regression line is shown for each analysis, the coefficient (gradient) is given in the right upper corner of each diagram. None of the data sets shown here has a linear distribution, and this was also the case for the other data sets in the analysis (data not shown).

Because no clear bimodal distribution could be detected in the fungal data sets, it was tested whether gene expression distribution in fungi might better be described by a distribution following Zipf’s law that was found in previous analyses of SAGE and microarray data from several species including yeast [1,5]. To analyze this, locus tags for each individual RNA-seq experiment were sorted by log_2_-transformed coverage, and the log_2_-transformed coverage was plotted against the log_2_-transformed rank after the sorting (Figure 3, Additional file 3: Method S2). In order to follow Zipf’s law, the resulting plots should give a linear distribution. However, this was not the case in any of the analyzed data sets, although the two yeasts *S. pombe* and *S. cerevisiae* displayed linearity over the middle part of the distribution curves (Figure 3). This is consistent with prior analyzes of SAGE data [1] and might indicate that over a certain range of expression levels, a power law distribution like Zipf’s law might apply, with the greater dynamic range of RNA-seq revealing deviations from this distribution in the tails. However, distributions from RNA-seq experiments of filamentous fungi do not show any linearity in the corresponding plots, indicating that they cannot be described by Zipf’s law.

## Conclusions

In summary, an analysis of 60 RNA-seq data sets from seven different fungi did not reveal a common distribution of global gene expression patterns. One caveat might be that in a number of studies mycelia containing different cell types were used; such a mixture of cell types might obscure distribution patterns. However, this hypothesis cannot explain short-term changes of distribution in otherwise identical samples, e.g. in *N. crassa*. Thus, it might seem possible that fungi can fine-tune their gene expression at the level of transcription not only for individual genes or chromosomal loci, but also at a global level. Another explanation might be that the distribution of peaks of gene expression itself evolves rather quickly, and therefore might be different in fungi when compared to metazoa, or even different between fungal species. However, the evolution of gene expression is not well understood yet, and therefore further studies involving more species and a wide variety of environmental conditions might be necessary to elucidate these processes [21]. So far, the analyses included only ascomycetes and one basidiomycete. Once data sets from early-diverging fungi, e.g. from Mucormycotina and chytrids, become available, it might be possible to draw conclusions on whether the bimodal distribution observed in metazoa [9] evolved only in this phylogenetic group or was present in the ancestor of animals and fungi, but lost or modified in the asco- and basidiomycetes.

## Methods

### Data sets

RNA-seq data sets downloaded from ArrayExpress (http://www.ebi.ac.uk/arrayexpress/), GEO (Gene Expression Omnibus, http://www.ncbi.nlm.nih.gov/geo/), and the Sequence Read Archive (SRA, http://www.ncbi.nlm.nih.gov/sra) are given in Additional file 1.

### Sequence read preparation, mapping, and counting

Data sets in .sra format were unpacked with the SRA Toolkit version 2.3.2. Sequence reads were analyzed and quality-trimmed with custom-made Perl scripts (available at http://c4-1-8.serverhosting.rub.de/public/) as described previously [11]. Reads of at least 40 bases after trimming were used for mapping, with the exception of the very short *S. pombe* reads (< 40 bases) that were not trimmed and directly used for mapping. For mapping, annotated mRNAs (or coding sequences for species where no mRNAs were annotated) were extracted from genome versions for the respective species as indicated in Additional file 1 using custom-made Perl scripts based on BioPerl [22]. Reads were mapped to annotated mRNAs or coding sequences with Tophat 2.0.8b [23] using Bowtie 2.1.0 [24] and SAMtools 0.1.19 [25]. Mapped reads were analyzed using custom-made Perl scripts. Only reads that mapped completely to a single locus tag were used in downstream analyses. For each mapped read, each base in the read was counted, yielding the number of bases mapped to each locus tag (coverage). Coverages were normalized within the data sets for each species to the length of the respective locus tag (mRNA or CDS length) and the number of bases counted for all locus tags for each RNA-seq experiment.

In previous experiments by Hebenstreit et al. [9], RPKM values were used as expression measurements instead of base coverage that was used in this study; and in previous analyses with the fungi *S. macrospora*[11] and *P. confluens*[12], mapping was performed against the reference genome instead of the annotated mRNAs as was done in this analysis. To exclude the possibility that these methodical differences change the shape of the distribution of expression levels, sequence reads from the *P. confluens* experiment GSM1020390 (dark grown vegetative mycelium, experiment DD1 from [12]) were mapped to the genome (data from [12]) or the annotated mRNAs (this analysis), and base coverage and RPKM values were calculated from the corresponding SAM files using custom-made Perl scripts. Histograms and kernel densities were plotted in R (Additional file 3: Figure S14). Bandwidth for density estimates were default values as described in [9]. The shapes of both histograms and kernel density estimates were preserved in all cases (Additional file 3: Figure S14) demonstrating that the distribution of gene expression levels is robust even with different gene expression measurements. Similar findings were also described by Hebenstreit et al. [9] who found that distributions were robust as long as log-transformed expression measurements were used for both RNA-seq and microarray data, and the bin-size for histograms and bandwidth for density estimates were small enough to preserve individual peaks.

### Clustering and/or curve fitting

Clustering by expectation-maximization was performed on the log_2_-transformed data using the mclust library [26] in R version 2.12.2 as described [9,11,12]. An example R command set used for clustering and plotting of data can be found in Additional file 3: Method S1.

### Test for distribution according to Zipf’s law

Each data set was sorted independently by normalized, log_2_-transformed expression, and log_2_-transformed expression was plotted against the log_2_-transformed expression rank for each locus tag. A least square linear regression line was calculated for each data set in R version 2.12.2. An example R command set used can be foundin Additional file 3: Method S2.

## Competing interests

The author declares that there are no competing interests.

## Supplementary Material

Additional file 1 Contains an overview of the RNA-seq studies that were used for the analysis of genome-wide transcript levels. Click here for file

Additional file 2 **Contains normalized, log**_**2**_**-transformed base counts for all data sets that were analyzed in this study.** Click here for file

Additional file 3 **Contains the following supplemental Figures, Tables and Methods:Figures S1-S11.** Distributions of gene expression levels for all RNA-seq data sets that were analyzed in this study. **Figure S12.** Analysis of the number of peaks or main peaks depending on the number of counted bases. **Figure S13.** Analysis of the number of peaks or main peaks depending on the number of protein-coding genes or the genome size. **Figure S14.** Different methods of analysis preserve the shape of the distribution of gene expression levels. **Table S1.** Summary of clustering by expectation-maximization. **Method S1.** Example for R commands for clustering by expectation-maximization and plotting of curves. **Method S2.** Example for R commands for testing if distribution follows Zipf's law. Click here for file
