# Supplementary material for: Fungal gene expression levels do not display a common mode of distribution
Source: BMC Res Notes. 2013 Dec 28;6:559. doi: 10.1186/1756-0500-6-559 (PMC3877863; doi:10.1186/1756-0500-6-559)
Supplement: Additional file 3 — Contains the following supplemental Figures, Tables and Methods:Figures S1-S11. Distributions of gene expression levels for all RNA-seq data sets that were analyzed in this study. Figure S12. Analysis of the number of peaks or main peaks depending on the number of counted bases. Figure S13. Analysis of the number of peaks or main peaks depending on the number of protein-coding genes or the genome size. Figure S14. Different methods of analysis preserve the shape of the distribution of gene expression levels. Table S1. Summary of clustering by expectation-maximization. Method S1. Example for R commands for clustering by expectation-maximization and plotting of curves. Method S2. Example for R commands for testing if distribution follows Zipf's law. [file 1756-0500-6-559-S3.pdf]

Additional file 3 (file name Additonal_file_3.pdf) contains the following supplemental Figures, Tables and Methods:

Figures S1-S11. Distributions of gene expression levels for all RNA-seq data sets that were analyzed in this study.

Figure S12. Analysis of the number of peaks or main peaks depending on the number of counted bases.

Figure S13. Analysis of the number of peaks or main peaks depending on the number of protein-coding genes or the genome size.

Figure S14. Different methods of analysis preserve the shape of the distribution of gene expression levels.

Table S1. Summary of clustering by expectation-maximization.

Method S1. Example for R commands for clustering by expectation-maximization and plotting of curves.

Method S2. Example for R commands for testing if distribution follows Zipf's law.

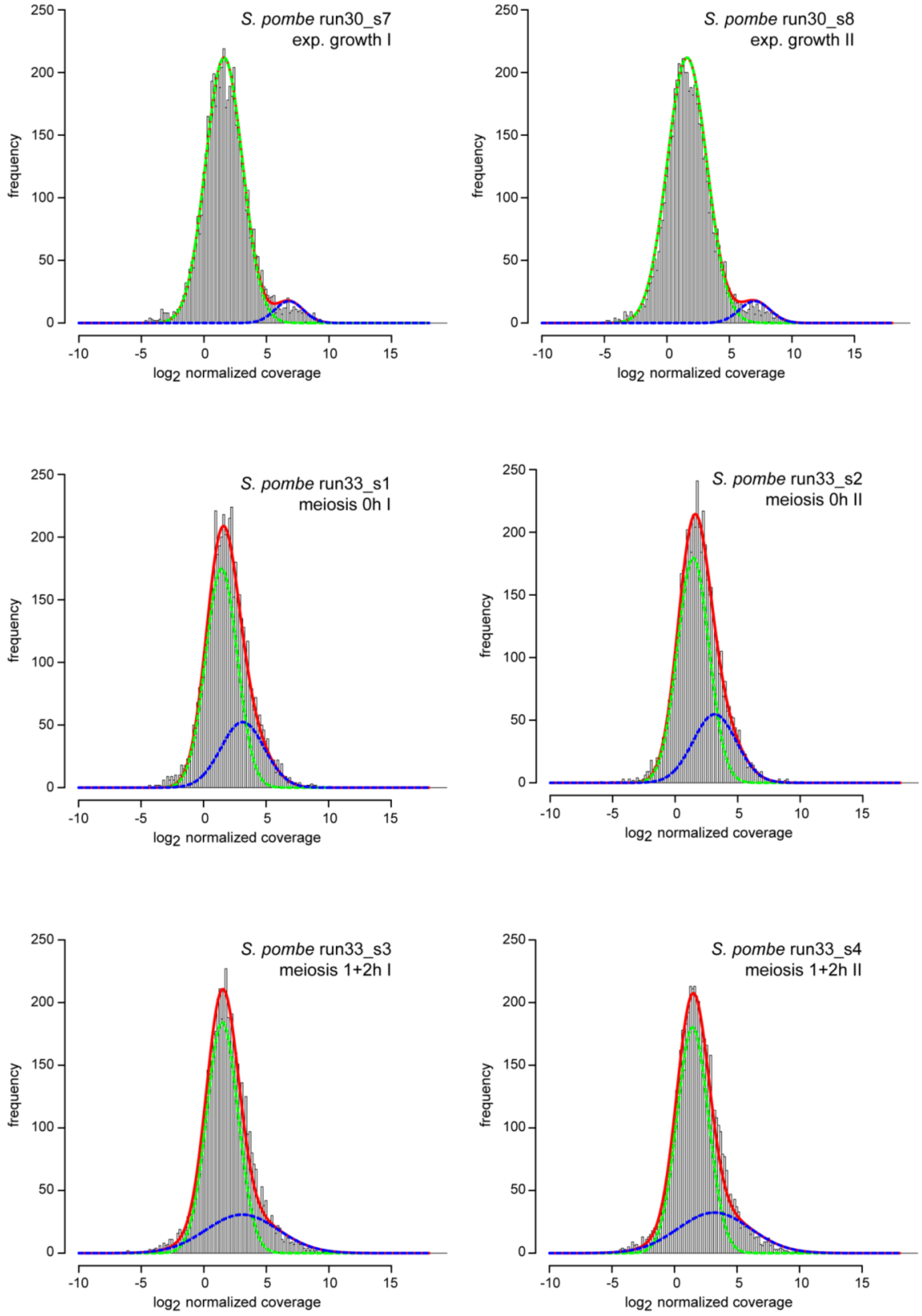

Figure S1. Distribution of gene expression levels for *Schizosaccharomyces pombe* RNA-seq data sets for exponential growth and meiosis (0-2 h). Histograms of normalized, $\log_2$-transformed coverage for each locus tag (grey bars), and estimated frequency distributions. Locus tags without coverage were not included in this analysis. The distribution function (red line) for each data set was dissected into components (blue and green lines) that are normal distributions with varying means and variances that make up different proportions of the observed distribution.

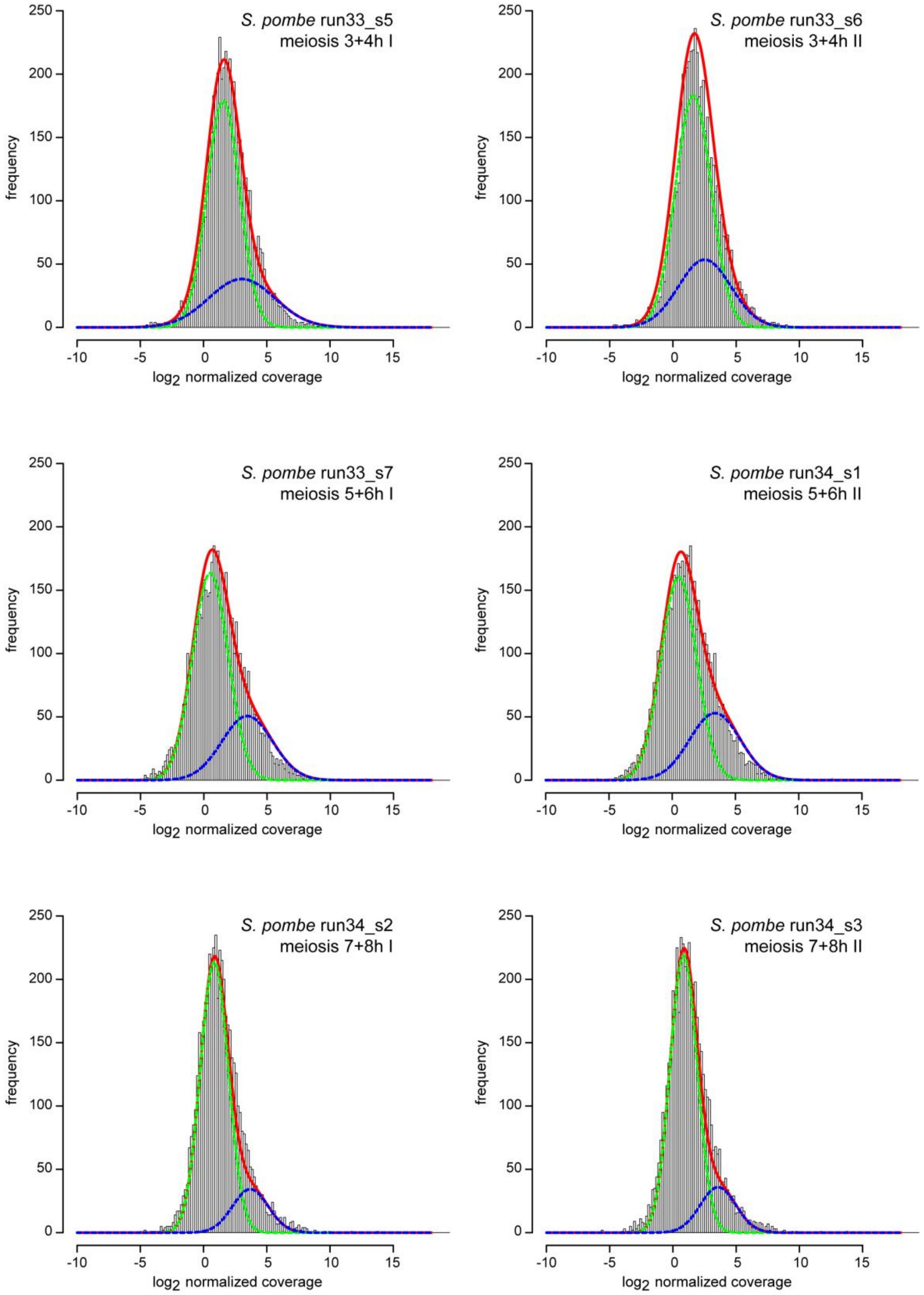

Figure S2. Distribution of gene expression levels for *Schizosaccharomyces pombe* RNA-seq data sets for meiosis (3-8 h). Histograms of normalized, $\log_2$-transformed coverage for each locus tag (grey bars), and estimated frequency distributions. Locus tags without coverage were not included in this analysis. The distribution function (red line) for each data set was dissected into components (blue and green lines) that are normal distributions with varying means and variances that make up different proportions of the observed distribution.

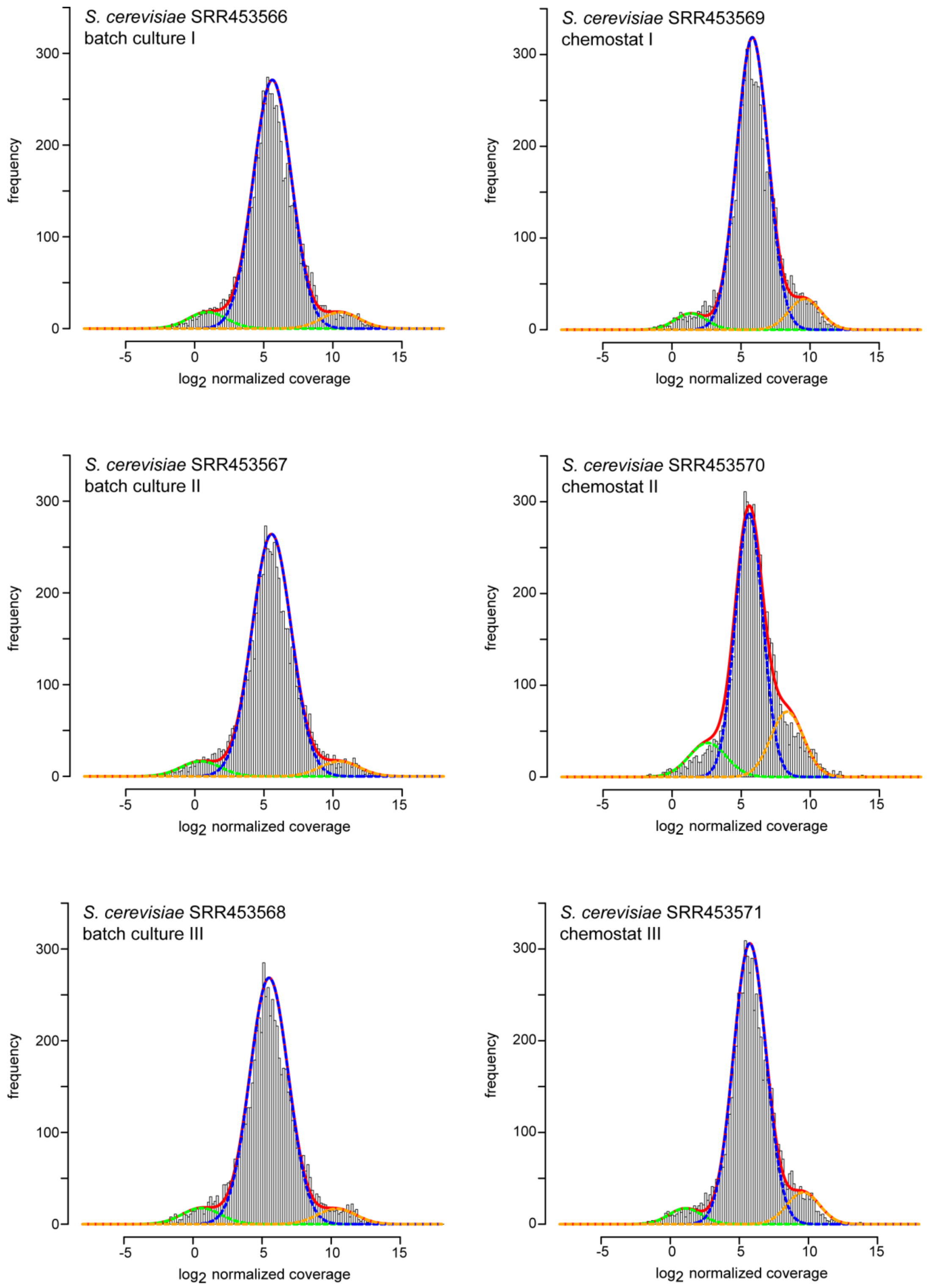

Figure S3. Distribution of gene expression levels for *Saccharomyces cerevisiae* RNA-seq data sets. Histograms of normalized, $\log_2$-transformed coverage for each locus tag (grey bars), and estimated frequency distributions. Locus tags without coverage were not included in this analysis. The distribution function (red line) for each data set was dissected into components (blue, green, and orange lines) that are normal distributions with varying means and variances that make up different proportions of the observed distribution.

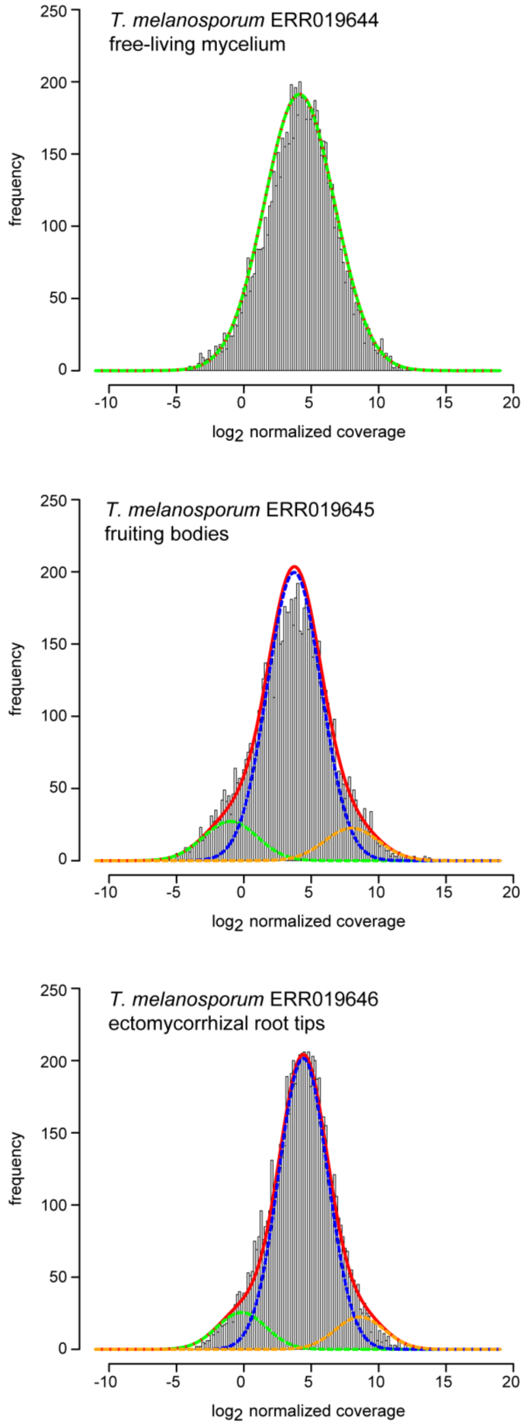

Figure S4. Distribution of gene expression levels for *Tuber melanosporum* RNA-seq data sets. Histograms of normalized, $\log_2$-transformed coverage for each locus tag (grey bars), and estimated frequency distributions. Locus tags without coverage were not included in this analysis. The distribution function (red line) for each data set was dissected into components (blue, green, and orange lines) that are normal distributions with varying means and variances that make up different proportions of the observed distribution.

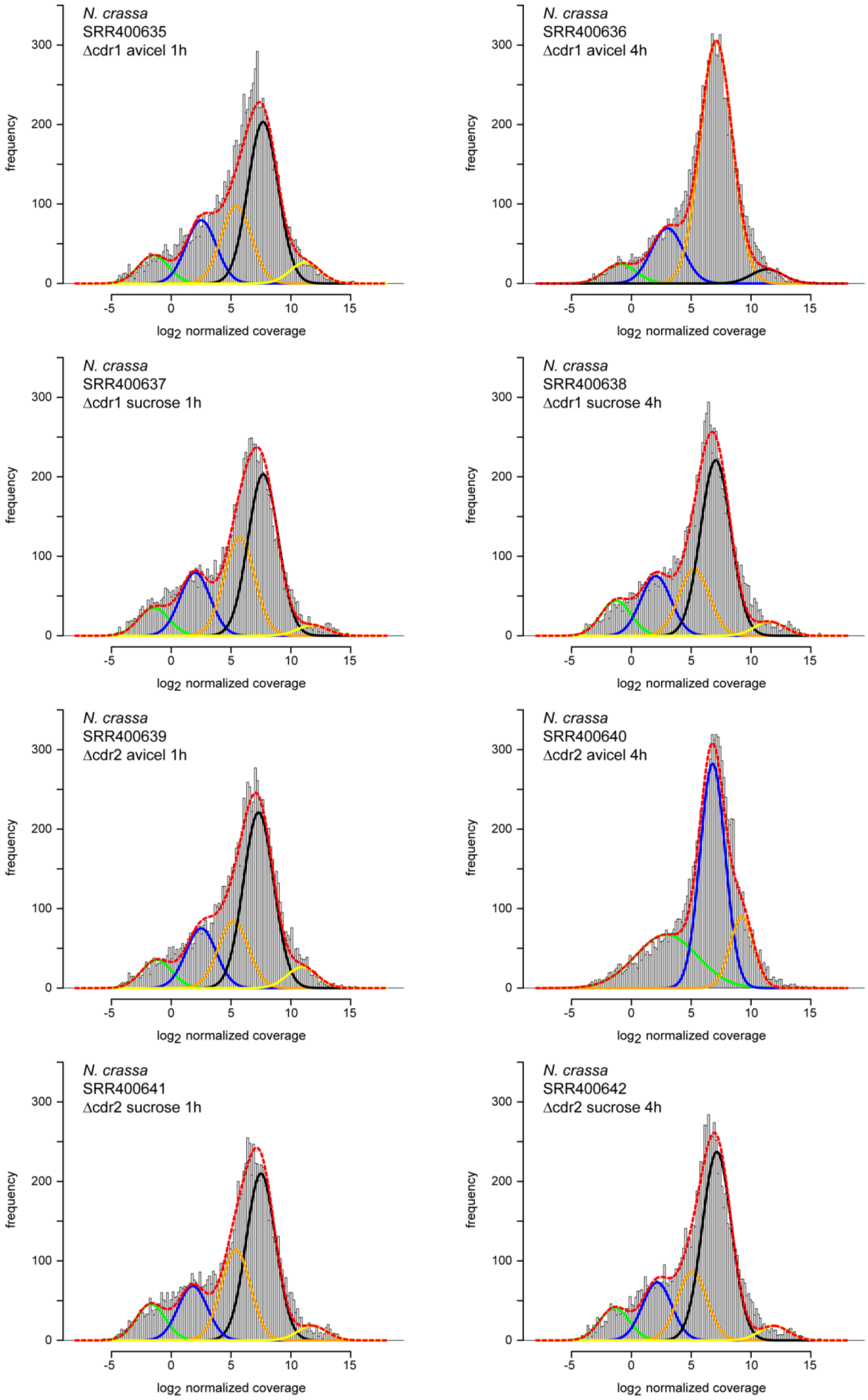

Figure S5. Distribution of gene expression levels for *Neurospora crassa* RNA-seq data sets for mutants Δcdr1 and Δcdr2. Histograms normalized, log_2-transformed coverage for each locus tag (grey bars), and estimated frequency distributions. Locus tags without coverage were not included in this analysis. The distribution function (red line) for each data set was dissected into components (blue, green, orange, yellow, and black lines) that are normal distributions with varying means and variances that make up different proportions of the observed distribution.

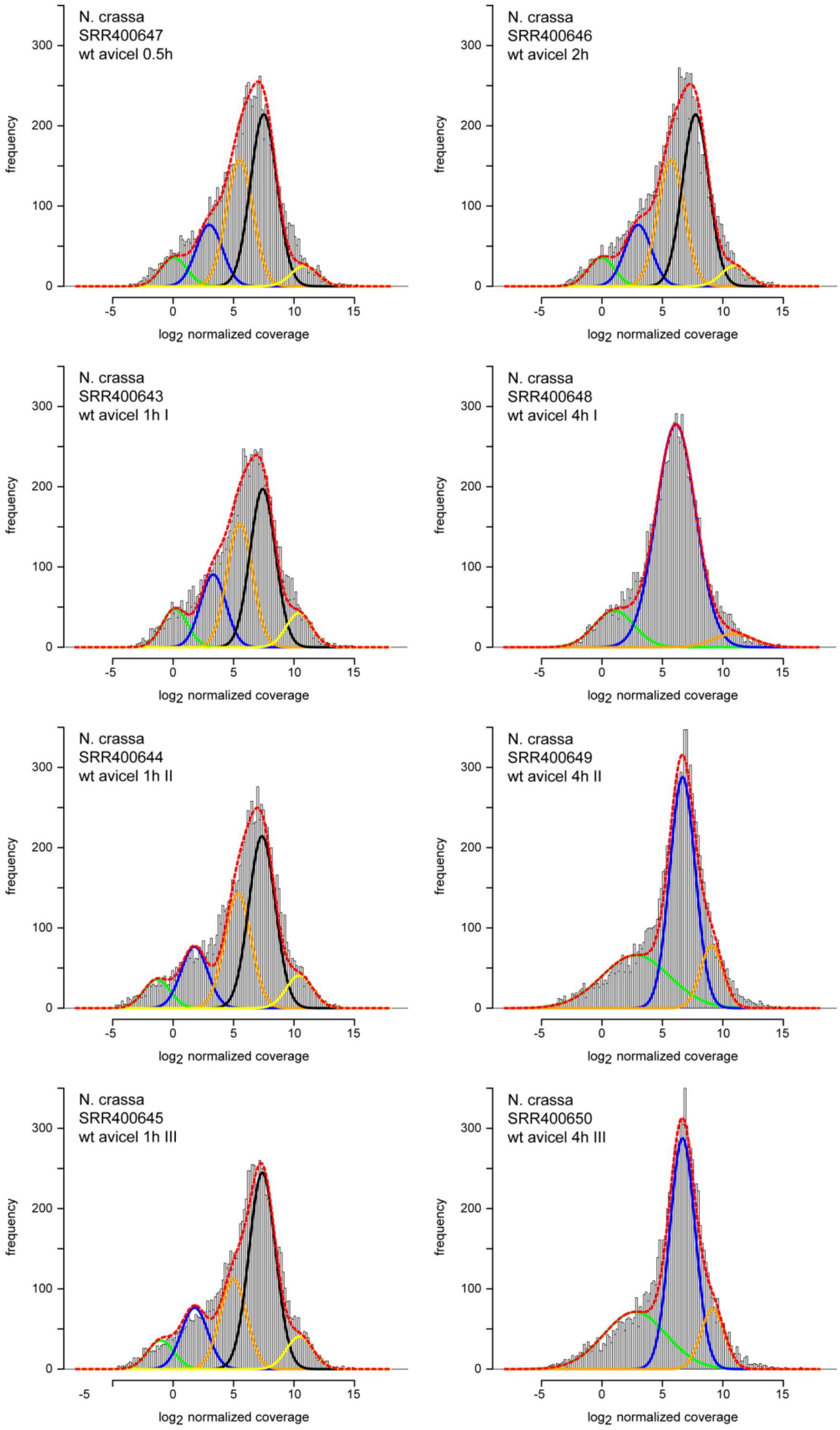

Figure S6. Distribution of gene expression levels for *Neurospora crassa* RNA-seq data sets for wild type grown on avicel. Histograms of normalized, $\log_2$-transformed coverage for each locus tag (grey bars), and estimated frequency distributions. Locus tags without coverage were not included in this analysis. The distribution function (red line) for each data set was dissected into components (blue, green, orange, yellow, and black lines) that are normal distributions with varying means and variances that make up different proportions of the observed distribution.

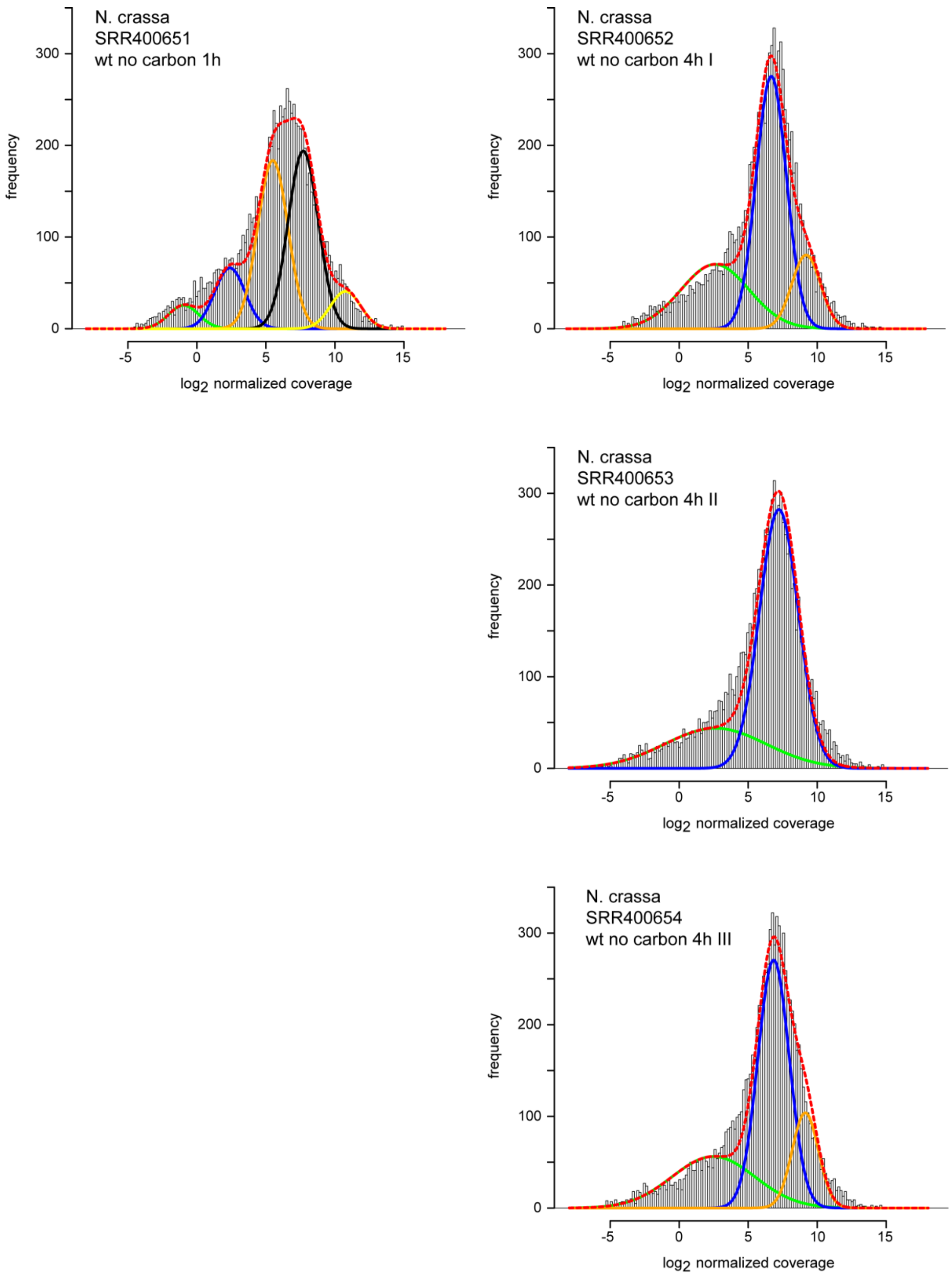

Figure S7. Distribution of gene expression levels for *Neurospora crassa* RNA-seq data sets for wild type grown without carbon source. Histograms of normalized, log_2-transformed coverage for each locus tag (grey bars), and estimated frequency distributions. Locus tags without coverage were not included in this analysis. The distribution function (red line) for each data set was dissected into components (blue, green, orange, yellow, and black lines) that are normal distributions with varying means and variances that make up different proportions of the observed distribution.

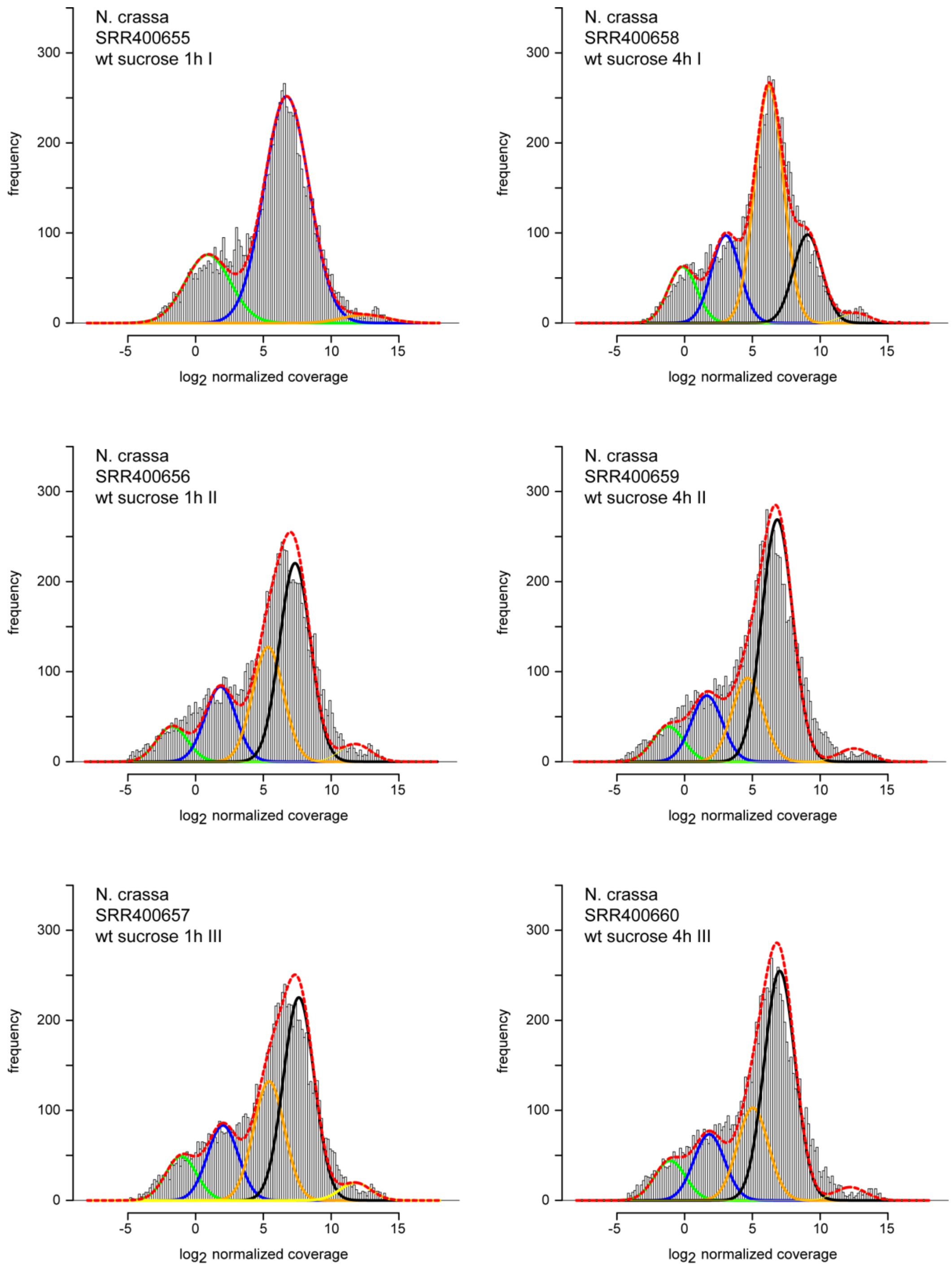

Figure S8. Distribution of gene expression levels for *Neurospora crassa* RNA-seq data sets for wild type grown on sucrose. Histograms of normalized, log_2-transformed coverage for each locus tag (grey bars), and estimated frequency distributions. Locus tags without coverage were not included in this analysis. The distribution function (red line) for each data set was dissected into components (blue, green, orange, yellow, and black lines) that are normal distributions with varying means and variances that make up different proportions of the observed distribution.

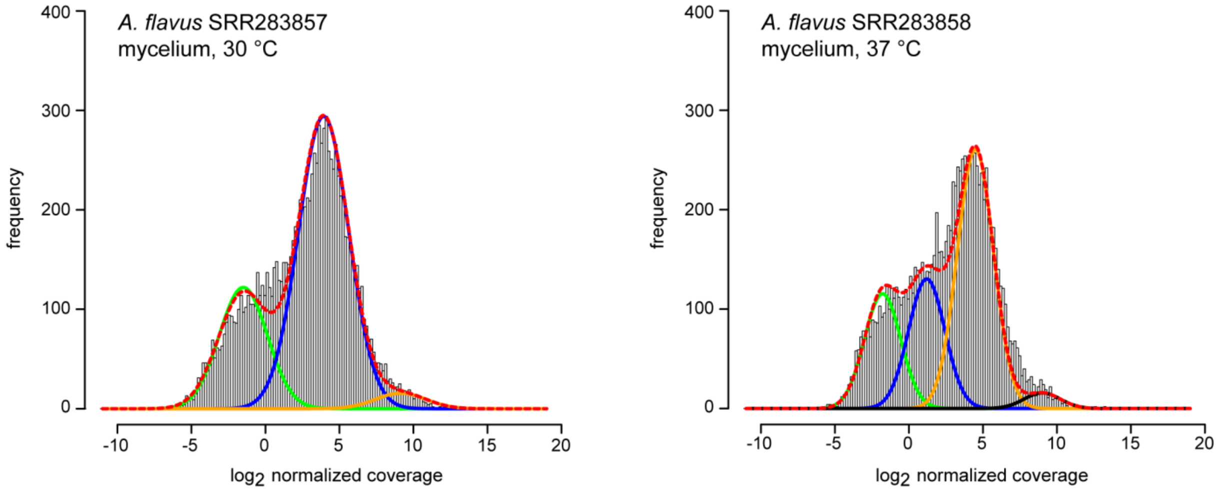

Figure S9. Distribution of gene expression levels for *Aspergillus flavus* RNA-seq data sets. Histograms of normalized, $\log_2$-transformed coverage for each locus tag (grey bars), and estimated frequency distributions. Locus tags without coverage were not included in this analysis. The distribution function (red line) for each data set was dissected into components (blue, green, orange, and black lines) that are normal distributions with varying means and variances that make up different proportions of the observed distribution.

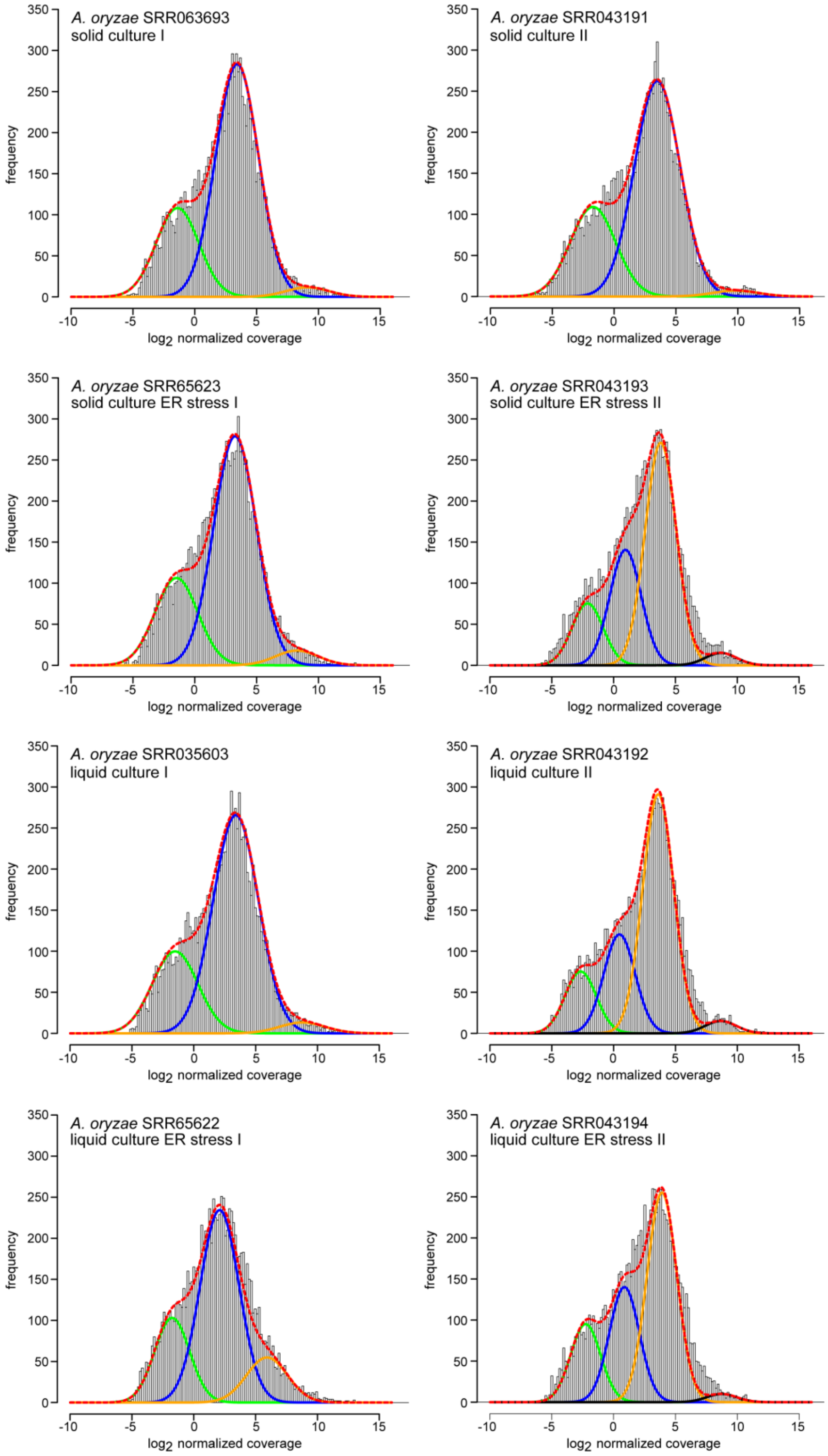

Figure S10. Distribution of gene expression levels for *Aspergillus oryzae* RNA-seq data sets. Histograms of normalized, $\log_2$-transformed coverage for each locus tag (grey bars), and estimated frequency distributions. Locus tags without coverage were not included in this analysis. The distribution function (red line) for each data set was dissected into components (blue, green, orange, and black lines) that are normal distributions with varying means and variances that make up different proportions of the observed distribution.

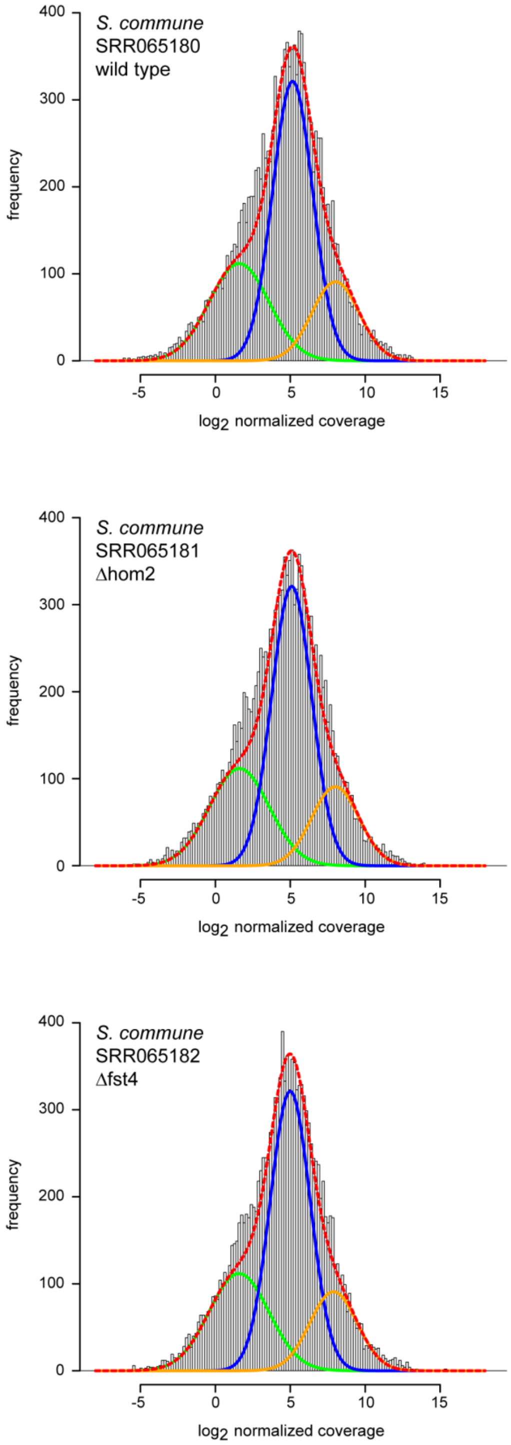

Figure S11. Distribution of gene expression levels for *Schizophyllum commune* RNA-seq data sets. Histograms of normalized, log_2-transformed coverage for each locus tag (grey bars), and estimated frequency distributions. Locus tags without coverage were not included in this analysis. The distribution function (red line) for each data set was dissected into components (blue, green, and orange lines) that are normal distributions with varying means and variances that make up different proportions of the observed distribution.

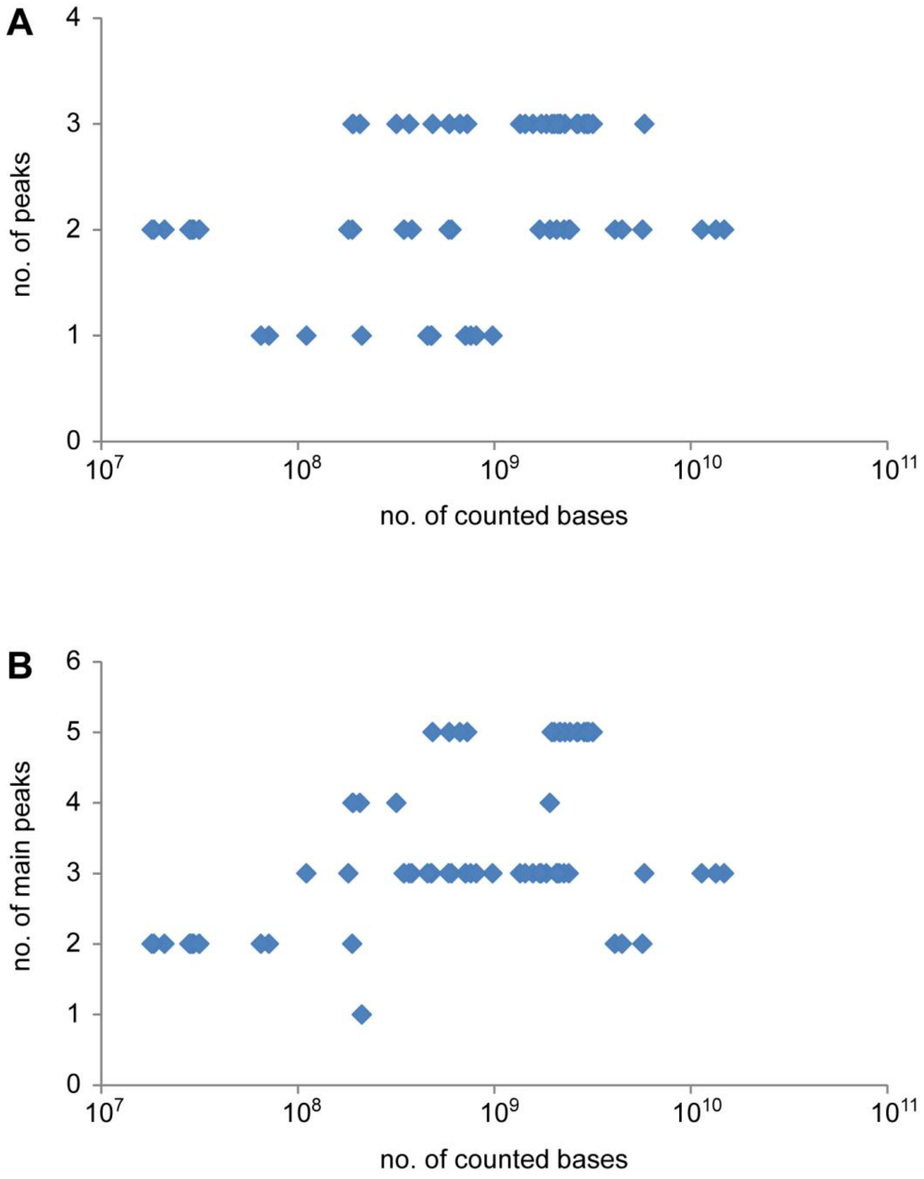

Figure S12. Analysis of the number of peaks (A) or main peaks (B) depending on the number of counted bases. No significant correlation exists between the number of peaks or main peaks and the number of counted bases in each RNA-seq data set.

A

species	genome size [Mb]	no. of protein-coding genes	min. no. of peaks	max. no. of peaks	min. no. of main peaks	max. no. of main peaks
S. pombe	12	5143	2	2	1	2
S. cerevisiae	12	6269	3	3	1	2
P. confluens	50	13369	3	3	2	2
T. melanosporum	125	7496	1	3	1	1
N. crassa	40	9730	2	5	1	3
S. macrospora	40	10091	2	3	1	3
A. flavus	37	13487	3	4	2	3
A. oryzae	37	12074	3	4	2	3
S. commune	39	14652	3	3	3	3

B

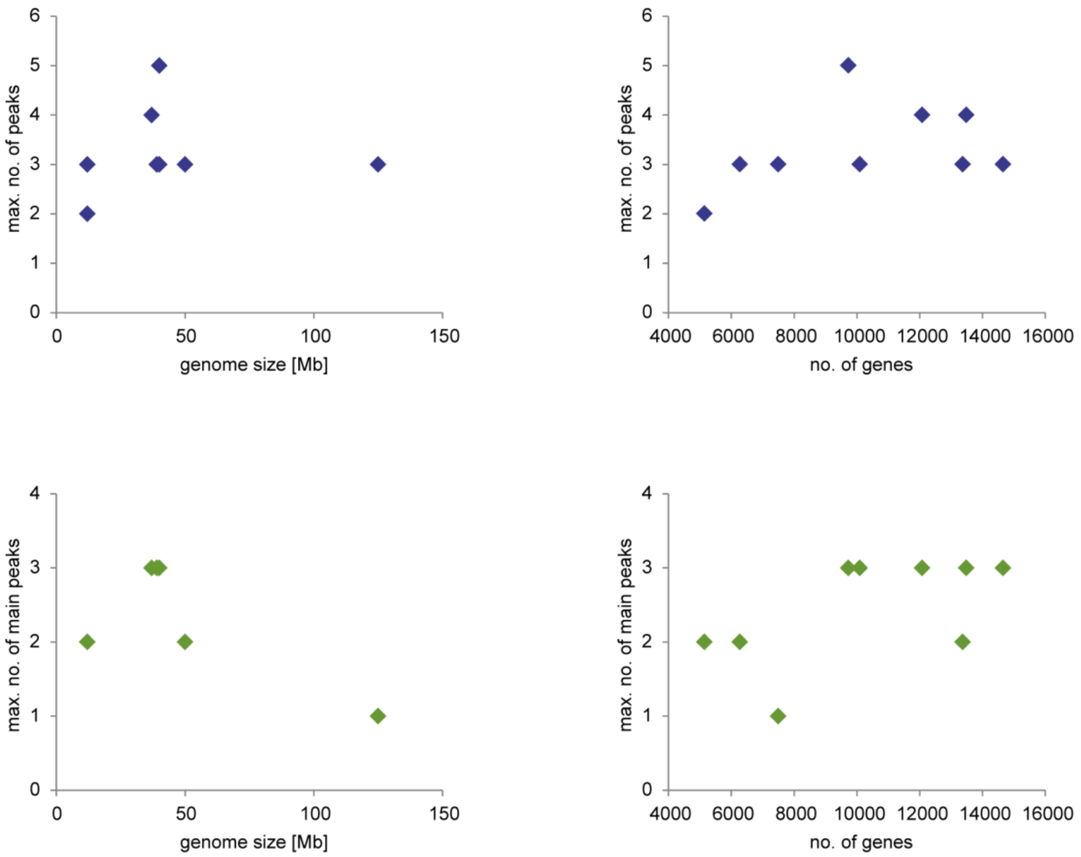

Figure S13. Analysis of the number of peaks or main peaks depending on the number of protein-coding genes or the genome size. (A) Summary of genome sizes and number of protein-coding genes in the annotation that was used for the analyses. (B) The number of peaks (top row) or main peaks (bottom row) was plotted against the genome size (left) or the number of genes (right). There is no clear correlation between the number of peaks or main peaks and the genome size or number of genes.

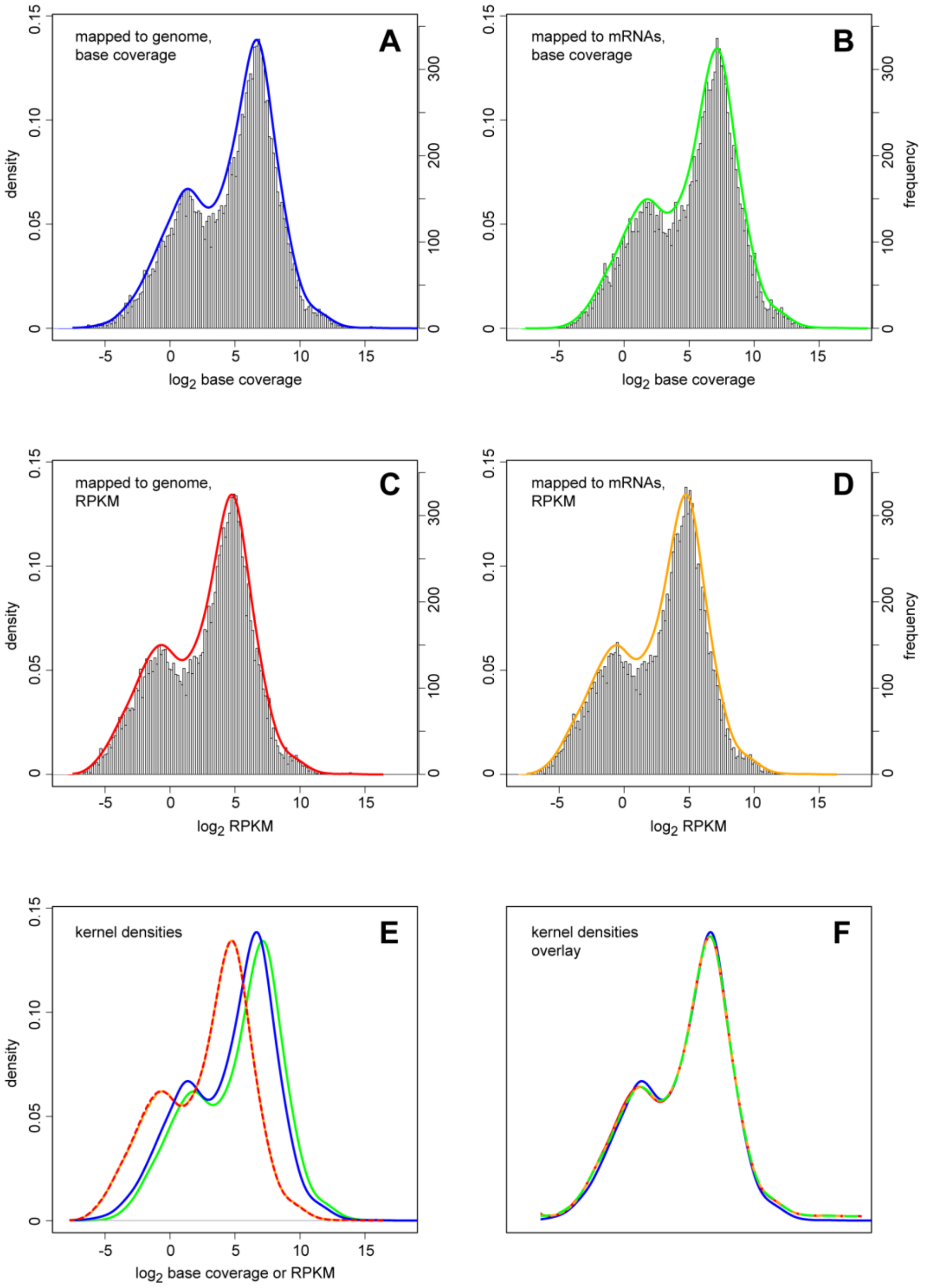

Figure S14. Different methods of analysis preserve the shape of the distribution of gene expression levels. Sequence reads from *P. confluens* experiment GSM1020390 (dark grown vegetative mycelium, experiment DD1 from Traeger et al. 2013) were mapped to the genome (data from Traeger et al. 2013) or the annotated mRNAs (this analysis), and base coverage and RPKM values, respectively, were calculated from the resulting SAM files using custom-made Perl scripts. (A-D) Histograms (frequency y-axes on the right) and kernel densities (y-axes on the left) were plotted in R. Bandwidth for density estimates were default values as described in Hebenstreit et al. 2011. (E) Kernel densities for all four analyses were plotted in one diagram. (F) Overlay of kernel densities shows that the overall shape of the distribution is preserved in all cases.

Table S1. Summary of clustering by expectation-maximization. Results for *P. confluens* and *S. macrospora* were from previous analyses (Teichert et al. 2012, Traeger et al. 2013), the other analyses were done in this study. Mean, variance and proportion are given for each of the components (peaks) into which each gene expression distribution could be dissected.

sample	no. of peaks	main peaks[1]	model[2]	component 1			component 2			component 3			component 4			component 5		
				mean	var.	prop.	mean	var.	prop.	mean	var.	prop.	mean	var.	prop.	mean	var.	prop.
S. pombe run30_s7	2	1	U	1.60	2.50	0.94	7.00	1.50	0.06	NA	NA	NA	NA	NA	NA	NA	NA	NA
S. pombe run30_s8	2	1	U	1.60	2.50	0.94	3.87	1.50	0.06	NA	NA	NA	NA	NA	NA	NA	NA	NA
S. pombe run33_s1	2	2	U	1.42	1.60	0.71	3.11	3.00	0.29	NA	NA	NA	NA	NA	NA	NA	NA	NA
S. pombe run33_s2	2	2	U	1.43	1.60	0.71	3.15	3.00	0.29	NA	NA	NA	NA	NA	NA	NA	NA	NA
S. pombe run33_s3	2	2	U	1.47	1.60	0.72	3.06	8.33	0.28	NA	NA	NA	NA	NA	NA	NA	NA	NA
S. pombe run33_s4	2	2	U	1.45	1.60	0.71	3.16	8.40	0.29	NA	NA	NA	NA	NA	NA	NA	NA	NA
S. pombe run33_s5	2	2	U	1.54	1.70	0.70	3.01	6.80	0.30	NA	NA	NA	NA	NA	NA	NA	NA	NA
S. pombe run33_s6	2	2	U	1.60	2.00	0.70	2.50	4.29	0.30	NA	NA	NA	NA	NA	NA	NA	NA	NA
S. pombe run33_s7	2	2	U	0.50	2.00	0.70	3.42	4.00	0.30	NA	NA	NA	NA	NA	NA	NA	NA	NA
S. pombe run34_s1	2	2	U	0.50	2.00	0.68	3.37	4.00	0.32	NA	NA	NA	NA	NA	NA	NA	NA	NA
S. pombe run34_s2	2	2	U	0.84	1.22	0.83	3.69	2.00	0.17	NA	NA	NA	NA	NA	NA	NA	NA	NA
S. pombe run34_s3	2	2	U	0.84	1.14	0.82	3.56	2.00	0.18	NA	NA	NA	NA	NA	NA	NA	NA	NA
S. cerevisiae SRR453566	3	1	E	0.90	1.90	0.06	5.62	1.90	0.88	10.52	1.90	0.06	NA	NA	NA	NA	NA	NA
S. cerevisiae SRR453567	3	1	E	0.41	3.00	0.06	5.56	3.00	0.89	10.46	3.00	0.05	NA	NA	NA	NA	NA	NA
S. cerevisiae SRR453568	3	1	E	0.59	1.95	0.06	5.51	1.95	0.89	10.38	1.95	0.05	NA	NA	NA	NA	NA	NA
S. cerevisiae SRR453569	3	1	E	1.35	1.31	0.05	5.82	1.31	0.86	9.60	1.31	0.09	NA	NA	NA	NA	NA	NA
S. cerevisiae SRR453570	3	2	U	2.56	1.82	0.12	5.57	1.00	0.68	8.32	1.41	0.20	NA	NA	NA	NA	NA	NA
S. cerevisiae SRR453571	3	1	E	1.08	1.40	0.05	5.75	1.40	0.85	9.61	1.40	0.10	NA	NA	NA	NA	NA	NA
P. confluens GSM1020388/89	3	2	U	3.60	2.50	0.43	7.44	1.30	0.46	10.06	3.00	0.10	NA	NA	NA	NA	NA	NA
P. confluens GSM1020390/91	3	2	U	1.16	5.32	0.44	6.56	1.80	0.47	9.26	3.10	0.09	NA	NA	NA	NA	NA	NA
P. confluens GSM1020392/93	3	2	U	1.83	5.53	0.45	7.12	1.70	0.48	10.03	3.00	0.07	NA	NA	NA	NA	NA	NA
T. melanosporum ERR019644	1	1	NA	4.12	6.80	1.00	NA	NA	NA	NA	NA	NA	NA	NA	NA	NA	NA	NA

T. melanosporum ERR019645	3	1	E	-1.00	4.00	0.11	3.74	4.00	0.80	8.00	4.00	0.09	NA	NA	NA	NA	NA	NA
T. melanosporum ERR019646	3	1	E	-0.17	3.30	0.10	4.41	3.30	0.81	8.61	3.30	0.09	NA	NA	NA	NA	NA	NA
N. crassa SRR400635	5	3	E	-1.49	1.60	0.08	2.50	1.60	0.18	5.44	1.60	0.22	7.67	1.60	0.46	11.20	1.60	0.06
N. crassa SRR400636	4	2	E	-0.98	1.80	0.06	2.96	1.80	0.17	7.05	1.80	0.73	11.30	1.80	0.04	NA	NA	NA
N. crassa SRR400637	5	3	E	-1.46	1.60	0.08	2.00	1.60	0.18	5.70	1.60	0.28	7.69	1.60	0.43	11.80	1.60	0.03
N. crassa SRR400638	5	3	E	-1.33	1.60	0.10	2.00	1.60	0.17	5.20	1.60	0.19	7.00	1.60	0.50	11.50	1.60	0.04
N. crassa SRR400639	5	3	E	-1.22	1.60	0.08	2.50	1.60	0.17	5.20	1.60	0.19	7.30	1.60	0.50	10.95	1.60	0.06
N. crassa SRR400640	3	3	U	2.88	6.63	0.31	6.75	1.10	0.53	9.20	0.99	0.16	NA	NA	NA	NA	NA	NA
N. crassa SRR400641	5	3	E	-1.71	1.50	0.10	1.80	1.50	0.15	5.40	1.50	0.25	7.50	1.50	0.46	11.78	1.50	0.04
N. crassa SRR400642	5	3	E	-1.46	1.50	0.09	2.10	1.50	0.16	5.03	1.50	0.19	7.10	1.50	0.52	11.87	1.50	0.04
N. crassa SRR400643	5	3	E	0.24	1.10	0.09	3.40	1.10	0.17	5.60	1.10	0.29	7.50	1.10	0.37	10.50	1.10	0.08
N. crassa SRR400644	5	3	E	-1.30	1.20	0.07	1.80	1.20	0.15	5.40	1.20	0.28	7.40	1.20	0.42	10.50	1.20	0.08
N. crassa SRR400645	5	3	E	-1.01	1.20	0.07	1.80	1.20	0.15	5.00	1.20	0.22	7.40	1.20	0.48	10.50	1.20	0.08
N. crassa SRR400646	5	3	E	-0.06	1.20	0.07	3.00	1.20	0.15	5.72	1.20	0.31	7.76	1.20	0.42	10.97	1.20	0.05
N. crassa SRR400647	5	3	E	0.06	1.20	0.07	3.00	1.20	0.15	5.50	1.20	0.31	7.50	1.20	0.42	10.85	1.20	0.05
N. crassa SRR400648	3	1	E	1.10	2.70	0.13	6.21	2.70	0.82	11.00	2.70	0.05	NA	NA	NA	NA	NA	NA
N. crassa SRR400649	3	2	U	2.84	7.80	0.33	6.71	1.10	0.54	9.12	0.88	0.13	NA	NA	NA	NA	NA	NA
N. crassa SRR400650	3	2	U	2.76	6.71	0.33	6.68	1.10	0.54	9.17	0.94	0.13	NA	NA	NA	NA	NA	NA
N. crassa SRR400651	5	2	E	-0.94	1.20	0.05	2.40	1.20	0.13	5.51	1.20	0.36	7.71	1.20	0.38	10.78	1.20	0.08
N. crassa SRR400652	3	3	U	2.76	6.10	0.31	6.83	1.20	0.54	9.36	1.09	0.15	NA	NA	NA	NA	NA	NA
N. crassa SRR400653	2	2	U	2.61	13.53	0.29	7.21	1.98	0.71	NA	NA	NA	NA	NA	NA	NA	NA	NA
N. crassa SRR400654	3	3	U	2.44	9.02	0.29	6.80	1.30	0.53	9.09	0.91	0.17	NA	NA	NA	NA	NA	NA
N. crassa SRR400655	3	2	E	0.91	2.75	0.23	6.72	2.75	0.75	12.47	2.75	0.02	NA	NA	NA	NA	NA	NA
N. crassa SRR400656	5	3	E	-1.57	1.30	0.08	2.00	1.30	0.17	5.50	1.30	0.26	7.50	1.30	0.45	11.94	1.30	0.04
N. crassa SRR400657	5	3	E	-1.08	1.30	0.10	2.00	1.30	0.17	5.40	1.30	0.27	7.60	1.30	0.46	11.77	1.30	0.04
N. crassa SRR400658	5	3	E	-0.15	1.10	0.12	3.04	1.10	0.18	6.23	1.10	0.50	9.09	1.10	0.18	12.61	1.10	0.02
N. crassa SRR400659	5	3	E	-1.05	1.30	0.08	1.80	1.30	0.15	4.80	1.30	0.19	7.00	1.30	0.55	12.66	1.30	0.03

N. crassa SRR400660	5	3	E	-1.09	1.30	0.09	1.80	1.30	0.15	5.02	1.30	0.21	7.00	1.30	0.52	12.20	1.30	0.03
S. macrospora GSM832529	2	2	U	2.80	2.00	0.43	5.40	2.10	0.57	NA	NA	NA	NA	NA	NA	NA	NA	NA
S. macrospora GSM832533	2	2	U	3.11	3.60	0.71	3.81	21.8	0.29	NA	NA	NA	NA	NA	NA	NA	NA	NA
S. macrospora GSM832530	3	1	E	0.54	2.19	0.05	4.72	2.19	0.91	9.88	2.19	0.04	NA	NA	NA	NA	NA	NA
S. macrospora GSM832534	2	2	U	1.38	15.00	0.40	4.21	3.50	0.60	NA	NA	NA	NA	NA	NA	NA	NA	NA
S. macrospora GSM832531	3	3	U	-0.77	4.01	0.30	3.55	2.50	0.50	6.81	2.15	0.20	NA	NA	NA	NA	NA	NA
S. macrospora GSM832532	3	3	U	-1.02	4.37	0.30	3.38	2.50	0.50	4.37	2.28	0.20	NA	NA	NA	NA	NA	NA
S. macrospora GSM832535	3	2	U	-0.80	3.79	0.30	3.93	3.00	0.63	7.53	1.96	0.07	NA	NA	NA	NA	NA	NA
S. macrospora GSM832536	3	2	U	-0.82	4.47	0.30	4.04	3.00	0.63	7.64	2.04	0.07	NA	NA	NA	NA	NA	NA
A. flavus SRR283857	3	2	E	-1.49	2.90	0.27	3.93	2.90	0.69	9.14	2.90	0.04	NA	NA	NA	NA	NA	NA
A. flavus SRR283858	4	3	E	-1.77	1.50	0.22	1.20	1.50	0.25	4.49	1.50	0.50	9.01	1.38	0.03	NA	NA	NA
A. oryzae SRR043191	3	2	E	-1.72	3.40	0.29	3.49	3.40	0.69	9.81	3.40	0.02	NA	NA	NA	NA	NA	NA
A. oryzae SRR043192	4	3	E	-2.68	1.60	0.15	0.46	1.60	0.24	3.60	1.60	0.58	8.56	1.60	0.03	NA	NA	NA
A. oryzae SRR043193	4	3	E	-2.12	1.60	0.15	0.95	1.60	0.28	3.80	1.60	0.54	8.67	1.60	0.03	NA	NA	NA
A. oryzae SRR043194	4	3	E	-2.27	1.47	0.19	0.86	1.47	0.28	3.90	1.47	0.51	8.73	1.47	0.02	NA	NA	NA
A. oryzae SRR035603	3	2	E	-1.52	3.40	0.26	3.39	3.40	0.70	8.77	3.40	0.04	NA	NA	NA	NA	NA	NA
A. oryzae SRR063693	3	2	E	-1.41	3.00	0.27	3.41	3.00	0.70	9.12	3.00	0.03	NA	NA	NA	NA	NA	NA
A. oryzae SRR065622	3	3	U	-1.76	2.00	0.24	2.08	2.50	0.61	5.91	2.76	0.15	NA	NA	NA	NA	NA	NA
A. oryzae SRR065623	3	2	E	-1.49	3.00	0.26	3.25	3.00	0.69	8.25	3.00	0.05	NA	NA	NA	NA	NA	NA
S. commune SRR065180	3	3	U	1.58	4.00	0.28	5.16	1.80	0.54	8.02	2.50	0.18	NA	NA	NA	NA	NA	NA
S. commune SRR065181	3	3	U	1.60	4.00	0.28	5.09	1.80	0.54	8.00	2.50	0.18	NA	NA	NA	NA	NA	NA
S. commune SRR065182	3	3	U	1.58	4.00	0.28	5.00	1.80	0.54	7.88	2.50	0.18	NA	NA	NA	NA	NA	NA

[1]proportion ≥ 0.15 (15 %), [2]E: equal variance, U: unequal variance

Method S1. Example for R commands for clustering by expectation-maximization (a) and plotting of curves (b). Analysis was performed on the normalized, log_2-transformed mapping results using the mclust package (Fraley and Raftery 2002, J Amer Stat Assoc, 97:611–631). The example is for a dataset with equal variance (modelName="E"), in the case of varying variance, the corresponding commands have to be set to "V" as the model.

a) clustering

```
coverage <- read.delim("current/log_transformed_data.txt", header=TRUE, dec=",")
v1<-0:200
v2<-v1 * 0.16
v3<-v2 - 10
hist(coverage$single_experiment_data, breaks=v3, freq=TRUE, xlim=c(-8,18), ylim=c(0,350))

sample<-coverage$single_experiment_data[coverage$single_experiment_data > -10]
library(mclust)
clust<-Mclust(sample)
clust
class<- clust$classification
odd<-seq(from=1,to=NROW(sample), by=2)
even<-seq(from=2,to=NROW(sample), by=2)
round(cv1EMtrain(data= sample[odd],labels= class[odd]), 3)
model<-mstep(modelName="E", data = sample[odd], z=unmap(class[odd]))
model$parameters
classmodel<-map(estep(modelName="E", data= sample, parameters=model$parameters)$z)

classError(classmodel[odd], class[odd])$errorRate
classError(classmodel[even], class[even])$errorRate
classError(classmodel, class)$errorRate
classError(classmodel, class)$misclassified
round(bicEMtrain(sample[odd], labels= class[odd]), 0)
samplebic<-mclustBIC(sample)
samplebic
samplebicmodel<-summary(samplebic, sample)
samplebicmodel
```

b) plotting of a dataset described by five normal distributions

```
x<-seq(from=-8, to=18, by=0.01)
x1<-dnorm(x, model$parameters$mean[1], sqrt(model$parameters$variance$sigmasq[1]))
x2<-dnorm(x, model$parameters$mean[2], sqrt(model$parameters$variance$sigmasq[1]))
x3<-dnorm(x, model$parameters$mean[3], sqrt(model$parameters$variance$sigmasq[1]))
```

```
x4<-dnorm(x, model$parameters$mean[4],
sqrt(model$parameters$variance$sigmasq[1]))
x5<-dnorm(x, model$parameters$mean[5],
sqrt(model$parameters$variance$sigmasq[1]))
xsumnorm<-(x1*model$parameters$pro[1])+(x2*model$parameters$pro[2])+
(x3*model$parameters$pro[3])+(x4*model$parameters$pro[4])+(x5*model$paramet
ers$pro[5])
plot(xsumnorm, type="l", col="red", ylim=c(0, 0.25))
par(new="TRUE")
hist(sample, breaks=v3, freq=TRUE, xlim=c(-8, 18), ylim=c(0,350))
x1norm<-x1*model$parameters$pro[1]
x2norm<-x2*model$parameters$pro[2]
x3norm<-x3*model$parameters$pro[3]
x4norm<-x4*model$parameters$pro[4]
x5norm<-x5*model$parameters$pro[5]
par(new="TRUE")
plot(x1norm, type="l", col="green", ylim=c(0, 0.25))
par(new="TRUE")
plot(x2norm, type="l", col="blue", ylim=c(0, 0.25))
par(new="TRUE")
plot(x3norm, type="l", col="orange", ylim=c(0, 0.25))
par(new="TRUE")
plot(x4norm, type="l", col="black", ylim=c(0, 0.25))
par(new="TRUE")
plot(x5norm, type="l", col="yellow", ylim=c(0, 0.25))
```

Method S2. Example for R commands for testing if distribution follows Zipf's law. Analysis was performed on the normalized log_2-transformed mapping results.

```
data <- read.delim("current/log_transformed_data.txt", header=TRUE,
dec=",")
run <- data$single_experiment_data[data$single_experiment_data > -10]
run <- sort(run, decreasing=TRUE)
rank <- log2(1:(length(run)))
regression <- lsfit(rank, run)
intercept <- regression$coefficients[1]
gradient <- regression$coefficients[2]
plot(rank, run, main = c("single_experiment", intercept, gradient))
abline(a=intercept, b=gradient, col="red")
```
